# Attitudes toward posthumous assisted reproduction in China: a multi-dimensional survey

**DOI:** 10.1186/s12978-022-01423-9

**Published:** 2022-05-21

**Authors:** Jiliang Huang, Jue Li, Wanfen Xiao, Zhiling Li

**Affiliations:** 1 Reproductive Center of the First Affiliated Hospital of Shantou University Medical College, No. 57 Changping Road, Jinping, Shantou, 515041 Guangdong People’s Republic of China; 2 Department of Nursing, Shenzhen Hospital, Southern Medical University, Shenzhen, 518110 Guangdong People’s Republic of China

**Keywords:** Posthumous assisted reproduction, Embryos, Attitude, Offspring, Ethics

## Abstract

**Background:**

Professional legislation and ethics guidelines for posthumous assisted reproduction (PAR) are lacking in China. This study aims to measure the attitudes of the general public, IVF couples, and assisted reproductive technology (ART) practitioners toward PAR in China.

**Methods:**

A multi-dimensional survey was designed, and electronic questionnaires were used. General demographic data, reproductive viewpoints, attitudes toward PAR, interactive ability to predict the partner’s attitude toward PAR, and the legal attributes and rights to the disposal of posthumous embryos were evaluated.

**Results:**

The study found that the traditional Chinese viewpoints of fertility had changed. The approval rates for PAR were 79.10%, 55.32%, and 58.89%, in the general public, IVF couples, and ART practitioners, respectively. Most participants agreed that the psychological well-being of offspring should be previously considered before making a PAR decision (81.84%, 73.61%, and 76.98%, respectively). Multivariable logistic regression analysis showed that age, marital status, and gender were common influencing factors, while occupation, religion, and pregnancy history showed no influence on support for PAR. Males and females showed similar predictive abilities for their partners’ attitudes toward PAR (57.87% for males, 61.12% for females). Intracouple agreement analysis showed that the consistent rate of consistency in attitudes toward PAR was 65.28%.

**Conclusion:**

The findings suggested that the approval rate of PAR was relatively high in China. Legislation and ethics guidelines for PAR may be considered in China. The psychological well-being of offspring should be considered before the implementation of PAR. Due to the very large regional and demographic differences in China, investigation of a larger samples of participants is necessary.

**Supplementary Information:**

The online version contains supplementary material available at 10.1186/s12978-022-01423-9.

## Plain language summary

This study is based on the dilemma of how to deal with the remaining frozen embryos when a family structure changes (such as the accidental death of one or both partners). In this research, we systematically investigated the basic attitudes of different groups toward PAR, the consistency and prediction accuracy of attitudes between couples and their ability to predict their partners’ attitudes, and the balance between offspring well-being and reproduction through a multi-dimensional cross-sectional survey in China. Our study illustrated that the approval rates of PAR were relatively high among the public, IVF couples and ART practitioners. Couples’ attitude prediction accuracy and the intercouple concordance were moderate. The psychological well-being of offspring should be considered before the implementation of PAR. Moreover, an appropriate legal policy or specialized guidance for PAR may be considered and published in China. This research provides some advice and evidence for medical professionals and policymakers regarding practice and policymaking related to PAR. We also believe that this manuscript is valuable and helpful for all the researchers who are interested in the posthumous reproduction, not only in China.

**Supplementary Information:**

The online version contains supplementary material available at 10.1186/s12978-022-01423-9.

## Introduction

In recent years, the using of assisted reproductive technology (ART) has become a common and effective treatment for couples with infertility. With the advanced embryo culture technology and the increasing pregnancy rate (PR), an increasing number of embryos are frozen in reproductive centers. For embryo cryopreservation, ethical and legal problems have emerged. The question of how to deal with the remaining frozen embryos becomes complicated and challenging when a family structure changes (such as an accidental death). Therefore, posthumous assisted reproduction (PAR) was chosen as the main topic of this study.

PAR refers to using gametes or embryos to initiate conception after the death of a genetic parent [1, 2], which raises many controversial problems. Countries worldwide have different regulatory frameworks for posthumous reproduction. The complex ethical and legal issues have resulted in numerous countries—Canada, France, Germany, Norway, and Sweden—banning the procedure [3, 4]. However, certain countries such as the United States [5], Australia [6], and Israel [7], either allow it with some limitations or do not regulate it. Both the American Society for Reproductive Medicine (ASRM) and the European Society of Human Reproduction and Embryology (ESHRE) have discussed posthumous reproduction repeatedly. From 2004 to 2018, the ASRM reseased three editions [5, 8, 9] regarding issues related to PAR. In 2006, the ESHRE Task Force analyzed the ethical aspects of PAR and concluded that the partner’s posthumous reproduction was acceptable if the deceased person had given a written consent and a one-year minimum waiting period was required before a PAR treatment [10].

However, there is no relevant research or multi-dimensional study on the reproductive problems of Chinese people. More prominently, legislation and ethics guidelines are lacking. According to an administrative decree from the Ministry of Health of the People’s Republic of China, all reproductive centers are prohibited from providing assisted reproductive treatment (ART) to single women, and surrogacy is forbidden [11]. Therefore, after the death of a partner, the families of the deceased have to destroy the remaining frozen embryos, making the patients feel devastated and reproductive doctors feel so miserable. Research on attitudes toward the donation of frozen embryos in Chinese IVF patients has suggested that the couples are unwilling to donate or destroy embryos due to the emotional bonds with the embryos [12]. This manuscript seeks to highlight some core issues causing a clinical dilemma for reproductive doctors. The controversial questions are as follows: (I) What are the attitudes of the general public, IVF couples, and ART practitioners toward PAR? (II) What are the legal attributes of gametes and embryos? (III) Who can dispose of frozen embryos or gametes in the hospital when one of the members of the couple dies? (IV) Which should be considered first, the family inheritance or the healthy growth of offspring? (V) Can the will of the living spouse represent the will of the deceased?

Based on the above-mentioned questions, this study was designed to measure the attitudes of the general public, IVF couples, and ART practitioners toward PAR and provide more reasonable recommendations for improving the management frozen embryos.

## Materials and methods

Ethical approval was granted by the Ethics Committee of the First Affiliated Hospital of Shantou University Medical College (Approval No. SUMC-ER-R 2020009). Every participant was informed of the project purpose and read the Instructions for Participants (Additional file 1)*.* Participation was voluntary, and the participants were allowed to discontinue participation at any time. Only fully completed surveys were collected and analyzed, which helps to avoid data deletion.

### Questionnaire design

**Fig. 1:**
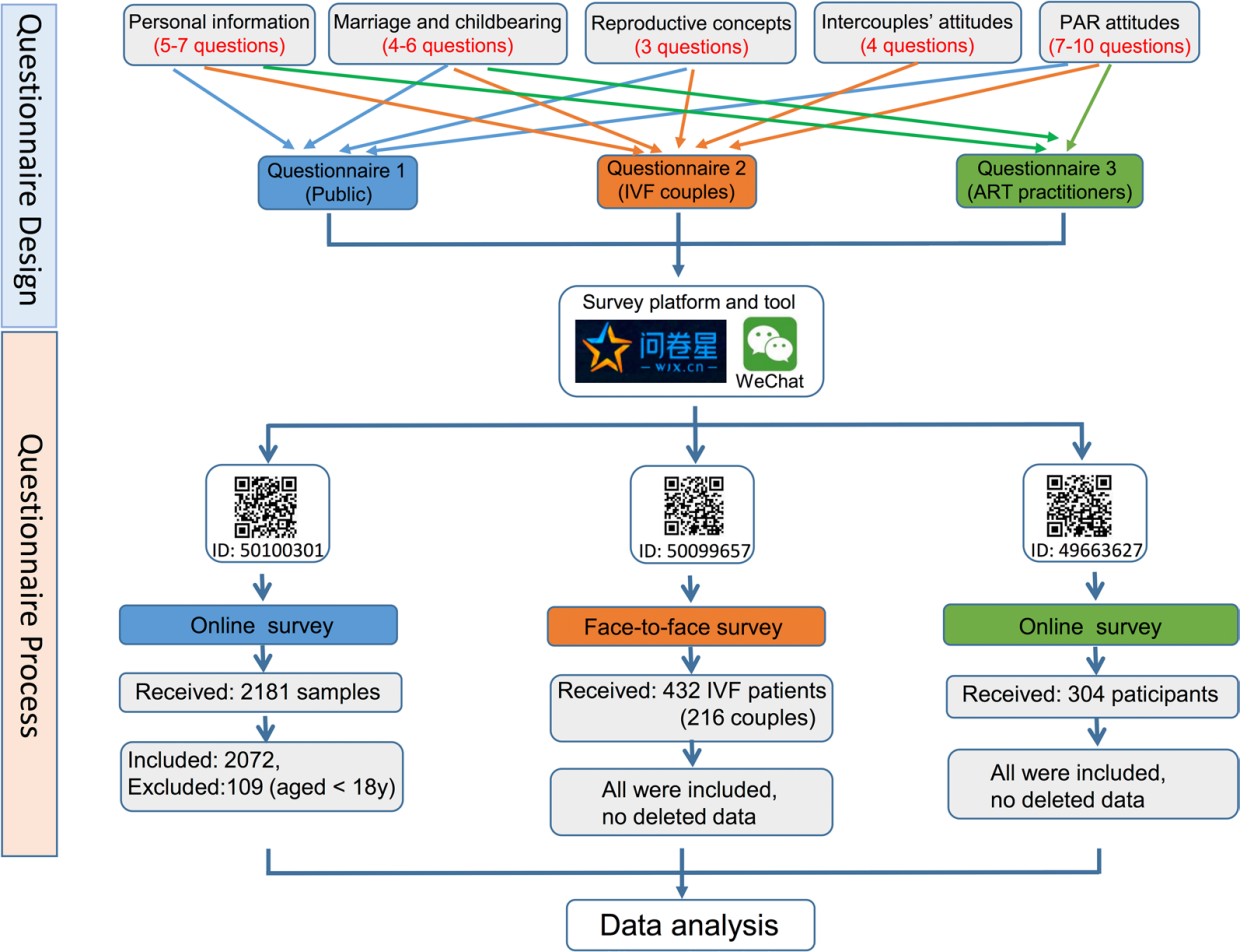
Flow chart of designing and processing the questionnaires

The flow chart is shown in Fig. 1. Three kinds of questionnaires were distributed, including a brief introduction for the participants, followed by 2–5 question sections to measure the attitudes of the general public, IVF couples, and ART practitioners toward PAR.

The first questionnaire was designed for the general public and consisted of four sections. The first section contained seven questions about the participants’ personal information to identify the personal influencing factors of their attitudes. The second section, including questions 8–12, mainly investigated the participants’ marital and fertility information, clarifying the attitudes and decision-making among groups with different pregnancy histories. The third section has three questions to investigate the participants’ reproductive viewpoints, thus clarifying whether traditional Chinese viewpoints have changed. The last ten questions in the fourth section were designed to examine the participants’ attitudes toward PAR. The first questionnaire in detail is attached as Additional file 2 (also available online: https://www.wjx.cn/jq/50100301.aspx).

The second questionnaire for the couples undergoing IVF was divided into five sections. In addition to the above four same sections in the first questionnaire, another section was added to investigate the consistency of attitudes between husbands and wives in decision-making and the ability of each spouse to predict decisions made by the other spouse. The second questionnaire in detail is attached as Additional file 3 (also available online: https://www.wjx.cn/jq/50099657.aspx).

The third questionnaire was designed for ART practitioners, and it includes personal information, marriage, fertility, and basic attitudes. The third questionnaire in detail is attached as Additional file 4 (also available online: https://www.wjx.cn/jq/49663627.aspx).

### Study protocol

Electronic questionnaires (e-questionnaire) were used in all three surveys, produced by the *Wenjuanxing* Survey System (https://www.wjx.cn), a professional online survey platform. A QR code was then generated and distributed via WeChat [13], the most frequently used social platform in China. WeChat has over 1.24 billion users, and 78% of people in China aged 16–64 use WeChat [14]. Therefore, the collection of questionnaires through WeChat could potentially reduce the sample bias and increase the representativeness of participants.

In the survey of the general public, adult Chinese people with no mental illness were the target population. Online surveys were used. Individuals aged 18 years and older were recruited online. There were no requirements for education levels, monthly incomes or marital status. To make the survey respondents more representative, we distributed the questionnaire in colleges, hospitals, government departments, supermarkets, communities, rural areas, and towns. Participants from different cities were recruited by friend groups in WeChat.

The survey of the IVF couples was conducted in the Reproductive Center, the First Affiliated Hospital of Shantou University Medical College. Infertile couples receiving IVF treatment were the only target population. Patients with ovulation induction or intrauterine insemination were excluded. In addition, patients who intended to receive the IVF treatment but had not yet entered the treatment cycle were not included in the study. The survey was completed when the couples visited doctors in person together. Only the face-to-face survey was used to identify the attitudes of IVF couples, considering the interactive attitude prediction within couples being one of the most important results. Our researchers stood beside the couples to prevent them from communicating with each other while completing the survey. A QR code was provided for the couples to complete the e-questionnaire.

To assess the attitudes of nationwide ART practitioners, most questionnaires were distributed during the 13th Annual Conference of the Chinese Society of Reproductive Medicine (CSRM). Some questionnaires were disseminated via friend groups in WeChat. Male specialists, female specialists, nurses, and embryologists were the target population. There were no restrictions for marital status, pregnancy history, or professional level.

### Statistical analysis

Data were analyzed by IBM SPSS Statistics (Version 22.0). Descriptive statistical analysis was applied to compare general demographic characteristics. Then, the Chi-square test was utilized to compare the intergroup differences, followed by multivariable logistic regression analysis for the influencing factors of PAR. An interrater agreement statistic was used to assess the accuracy of attitudes between spouses. The kappa index was evaluated. All reported *P* values were two-sided, and *P* < 0.05 was considered statistically significant.

## Results

### General demographic data

This study was conducted from November 2019 to February 2021. As shown in Fig. 1, 2181 members of the public completed the first questionnaire. Of those, 2072 participants were included in the analysis (response rate: 95%). A total of 109 subjects were excluded due to age. A total of 432 IVF patients and 304 ART practitioners completed in questionnaires 2 and 3, respectively, with no deleted data. The demographic characteristics of the participants are shown in Table 1, including gender, age, educational level, occupation, monthly income, religion, household registration, marital status, pregnancy history, and child numbers in each group. The survey was delayed for several months because of the outbreak of nCoV-2019. Additionally, some adjustments were applied according to different populations. For example, years of infertility was analyzed only in IVF couples, and professional levels were analyzed only in ART practitioners. The results showed that the mean ages were 30.77 ± 8.69 (aged 18–68) in the general public, 32.83 ± 4.63 (aged 23–47) in IVF couples, and 34.08 ± 8.09 (aged 20–56) in ART practitioners. More than half (58.96%) of the general public and 63.48% of ART practitioners within the subjects were married. The IP addresses of the participants covered more than 30 cities in 18 provinces among the general public (Additional file 5: Fig. S1) and 21 provinces among IVF practitioners (Additional file 6: Fig. S2). The sufficient and appropriate proportion of married reproductive-aged participants indicated that the participants’ primary demographic data could ensure the validity of this study.

**Table 1:** Demographic characteristics of participants

The general public (N = 2076)		IVF patients (N = 432)		ART practitioners (N = 304)	
Items	N (%)	Items	N (%)	Items	N (%)
*Gender*		*Age (y)*		*Age (y)*	
Male	644 (31.02%)	23–34	280 (64.81%)	20–29	96 (31.59%)
Female	1432 (68.98%)	35–44	152 (35.19%)	30–39	131 (43.09%)
*Age (y)*		*Education*		40–49	62 (20.39%)
18–24	509 (24.52%)	Below college	188 (43.52%)	≥ 50	15 (4.93%)
25–34	1058 (50.96%)	College	182 (42.13%)	*Education*	
35–44	309 (14.88%)	Post-graduate	62 (14.35%)	Bachelor&Below	139 (45.72%)
≥ 45	196 (9.44%)	*Occupation*		Master degree	126 (41.45%)
*Education*		Liberal work	51 (11.81%)	PHD degree	39 (12.83%)
Below college	270 (13.01%)	Business	138 (31.94%)	*Occupation*	
College	1300 (62.62%)	General staff	162 (37.50%)	Male-specialist	36 (11.84%)
Post-graduate	506 (24.37%)	Technical post	81 (18.75%)	Female-specialist	107 (35.20%)
*Occupation*		*Monthly income (¥)*		Nurse	91 (29.93%)
Liberal work	367 (17.68%)	≤ 3000	99 (22.92%)	Lab-technicians	70 (23.03%)
Business	120 (5.78%)	3001–6000	153 (35.42%)	*Professional level*	
General staff	414 (19.94%)	6001–9000	82 (18.98%)	Primary	95 (31.25%)
Technical post	1175 (56.60%)	≥ 9001	98 (22.68%)	Secondary	112 (36.84%)
*Monthly income (¥)*		*Religion*		Vice-senior	64 (21.05%)
≤ 3000	494 (23.80%)	Buddhism	120 (27.78%)	Senior	33 (10.86%)
3001–6000	485 (23.36%)	Christian	4 (0.93%)	*Marital status*	
6001–9000	455 (21.92%)	Others	10 (2.31%)	Married	193 (63.49%)
≥ 9001	642 (30.92%)	No	298 (68.98%)	Single	111 (35.51%)
*Religion*		*Registration*		*Pregnancy history*	
Buddhism	299 (14.40%)	Rural	272 (62.961%)	Yes	162 (53.29%)
Christian	48 (2.31%)	Urban	160 (37.04%)	No	142 (46.71%)
Others	65 (3.13%)	*Marital status*		*Conceived manner*	*(N = 162)*
No	1664 (80.15%)	First	375 (86.81%)	Nature	130 (80.25%)
*Registration*		Remarried	57 (13.19%)	ART	32 (19.75%)
Rural	757 (36.45%)	*Years of infertile*		*Children*	*(N = 162)*
Urban	1319 (63.54%)	≤ 1	27 (6.25%)	1	86 (53.09%)
*Marital status*		1–4	199 (46.07%)	≥ 2	55 (33.95%)
Married	1224 (58.96%)	4–7	156 (36.11%)	None	21 (12.96%)
Single	852 (41.04%)	≥ 7	50 (11.57%)		
*Marriage (y)*	*(N = 1224)*	*Pregnancy history*			
≤ 1	106 (8.66%)	Yes	196 (45.37%)		
2–4	275 (22.47%)	No	236 (54.63%)		
4–7	267 (21.81%)	*Conceived manner*	*(N = 196)*		
≥ 7	576 (47.06%)	Nature	106 (54.08%)		
*Pregnancy history*		ART	90 (45.92%)		
Yes	1067 (51.40%)	*Children*	*(N = 196)*		
No	1009 (48.60%)	1	83 (42.35%)		
*Conceived manner*	*(N = 1067)*	≥ 2	18 (9.18%)		
Nature	1040 (97.47%)	None	95 (48.47%)		
ART	27 (2.53%)				
*Children*	*(N = 1224)*				
1	546 (44.61%)				
≥ 2	378 (30.88%)				
None	300 (24.51%)				

### Traditional reproductive viewpoints

**Fig. 2:**
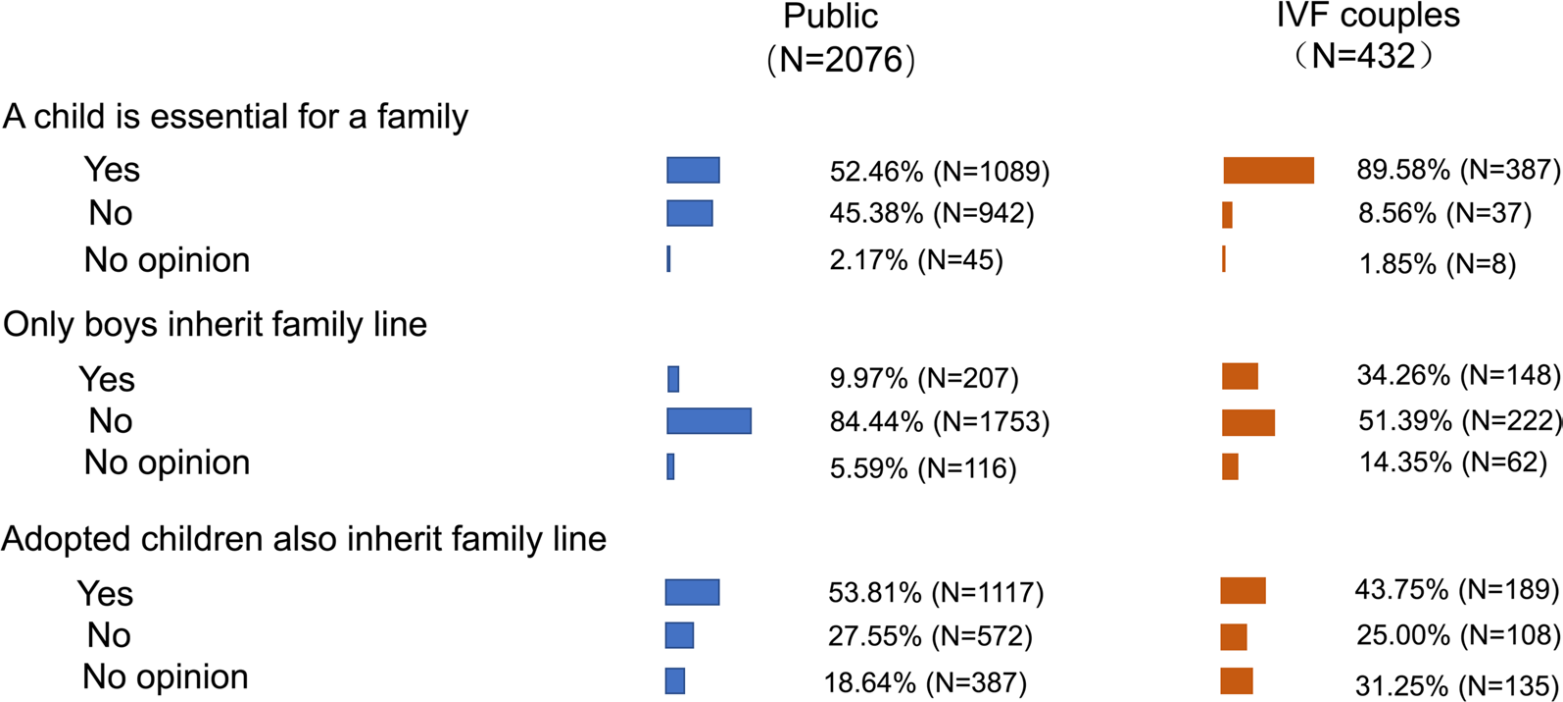
Traditional reproductive viewpoints between Public and IVF patients. The length of the color bar represented the percentage

Traditional reproductive attitudes of the general public and IVF couples were investigated (Fig. 2). Among the included participants, over half (52.46%) of the general public believed that children were essential for a family. This attitude was more commonly asserted in IVF couples (89.58%). Surprisingly, 1753 of 2076 participants from the general public (84.44%) believed that not only boys could pass on the family line, and this view was significantly lower in IVF patients (51.39%). More than half (53.81%) of the general public and 43.75% of IVF patients believed that adopted children can also pass on the family line. There was a significant inconsistency between the general public and the IVF couples regarding whether adopted children can also pass on the family line (*X*^2^ = 35.11, *P* = 0.000).

### Attitudes toward PAR-related issues

**Fig. 3:**
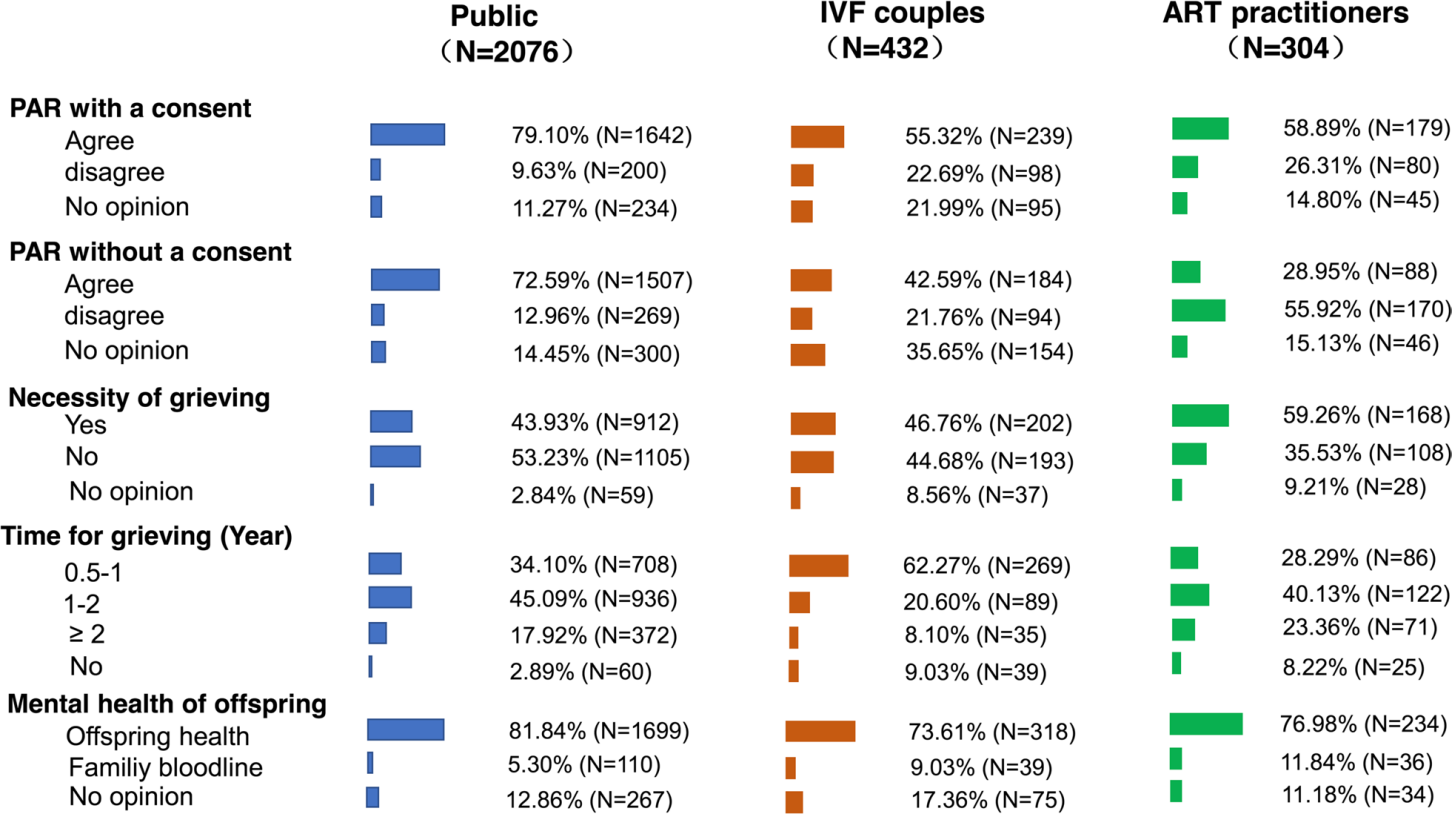
Attitudes toward PAR-related issues in public, IVF patients, and ART practitioners. The length of the color bar represents the percentage. *PAR* posthumous assisted reproduction. The complete sentence of the last question is “Inheriting family blood or ensuring offspring healthy grow-up, which do you think is more important?”

The attitudes of the general public, IVF patients, and ART practitioners toward PAR-related issues are shown in Fig. 3. All the three groups showed high approval rates for PAR when the will of the decreased was clear (79.10%, 55.32%, and 58.89%, respectively). Among the included participants, only portion of IVF patients (42.59%) and ART practitioners (28.95%) still supported PAR when the willingness of the deceased was unclear, which was significantly different from the opinion in the general public (72.59%, *P* = 0.000). Approximately half of the public participants (53.23%) and IVF couples (44.68%) thought it was unnecessary to allow adequate time for grieving and insisted on the right of the surviving spouse to choose to transplant the posthumous embryos. However, 168 of 304 ART practitioners (59.26%) thought it necessary to allow adequate grieving time. When it comes to the duration for grieving, more IVF couples (62.27%) tended to prefer a shorter grieving time (for 0–1 year) than the general public (34.10%) and ART practitioners (28.29%). Unsurprisingly, the attitudes of the three groups on the mental healthy growth of offspring were highly consistent (81.84% for the general public, 73.61% for IVF couples, and 76.98% for ART practitioners).

### Influencing factors of PAR-related issues

Multivariable logistic regression analysis was applied to identify the influencing factors of PAR in the three groups (Table 2). Among the included participants, the demographic characteristics associated with support for PAR were different in the three groups. However, age, marital status, and gender were common influencing factors. In the general public group, multivariable logistic regression analysis showed that younger age (OR 3.48 for below 34 years old), a married marital status (OR 3.46, 95% CI 1.28–9.36), and lower-income participants (OR 2.53 for monthly income below 3000 CNY and OR 1.75 for 3001–6000 CNY) had a positive relationships with support for PAR. However, the male participants (OR 0.54, 95% CI 0.38–0.77) and the less educated participants (OR 0.04, 95% CI 0.02–0.07 for those below college education) showed negative correlations with supporting PAR. Occupation, religion, household registration, pregnancy history, or number of children were not significantly correlated with attitudes toward PAR. In the IVF patients, similar significant positive relationships were was found in younger (OR 4.54 for below 34 years old), first married (OR 5.07, 95% CI 1.96–13.13) patients. A strong negative association appeared in males (OR 0.41, 95% CI 0.21–0.83), the less educated patients (OR 0.05, 95% CI 0.01–0.19 for those below college education), and more low-income patients (OR 0.27 for monthly income below 3000 CNY and OR 0.33 for 3001–6000 CNY). Occupation, religion, household registration, and pregnancy history did not influence the attitudes toward PAR. Although the gender of the practitioners was not investigated, the results indicated that female fertility specialists showed a higher support rate (OR 2.42, 95% CI 1.05–5.58). As with the results of the first two groups, higher supportive attitudes were found in younger and married practitioners (OR 4.83 and 3.80, respectively). IVF practitioners with a history of conception showed a stronger support (OR 7.18, 95% CI 1.67–31.12), which was different from the results of the previous two groups.

**Table 2:** Logistic regression predicting demographic characteristics associated with supporting PAR

		Public			IVF couples			ART practitioners		
		P	OR	95%CI	P	OR	95%CI	P	OR	95%CI
Gender								Not investigated		
Male		0.00^*^	0.54	0.38–0.77	0.01^*^	0.41	0.21–0.83			
Female		Referent			Referent					
Age group										
≤ 34		0.00^*^	3.48	1.76–6.90	0.01^*^	4.54	1.53–13.56	0.01^*^	4.83	1.55–15.07
≥ 35		Referent			Referent			Referent		
Education level								Education level^a^		
Below college		0.00^*^	0.04	0.02–0.07	0.00^*^	0.05	0.01–0.19	0.66	1.27	0.44–3.61
College degree		0.34	0.79	0.48–1.29	0.07	0.28	0.07–1.10	0.03^*^	3.18	1.10–9.16
Above college		Referent			Referent			Referent		
Occupation								Occupation^b^		
Liberal work		0.11	0.58	0.29–1.14	0.07	3.12	0.91–10.72	0.18	2.06	0.71–5.94
Business		0.92	1.03	0.54–1.97	0.24	1.75	0.68–4.45	0.04^*^	2.42	1.05–5.58
General staff		0.69	1.11	0.68–1.79	0.22	1.89	0.69–5.12	0.08	2.22	0.91–5.39
Technical post		Referent			Referent			Referent		
Monthly income (¥)								Professional level^c^		
≤ 3000		0.01^*^	2.53	1.28–5.03	0.04^*^	0.27	0.08–0.94	0.31	0.43	0.08–2.20
3001–6000		0.03^*^	1.75	1.05–2.93	0.03^*^	0.33	0.13–0.87	0.07	0.27	0.07–1.12
6001–9000		0.21	1.37	0.84–2.25	0.35	0.58	0.19–1.82	0.13	0.34	0.08–1.39
≥ 9001		Referent			Referent			Referent		
Religion								Not investigated		
Yes		0.06	1.53	0.98–2.39	0.09	1.92	0.91–4.04			
No		Referent			Referent					
House registration								Not investigated		
Rural		0.64	1.10	0.74–1.62	0.07	1.98	0.94–4.17			
Urban		Referent			Referent					
Marital status					Marital status^d^					
Married		0.01^*^	3.46	1.28–9.36	0.00^*^	5.07	1.96–13.13	0.01^*^	3.80	1.33–10.83
Single		Referent			Referent			Referent		
Pregnancy history										
Yes		0.41	0.56	0.14–2.23	0.56	0.70	0.21–2.30	0.01^*^	7.18	1.67–31.12
No		Referent			Referent			Referent		
Conceived manner										
Nature		0.03^*^	3.71	1.14–12.07	0.81	0.87	0.28–2.73	0.66	0.76	0.23–2.77
ART		Referent			Referent			Referent		
Children										
1		0.49	0.81	0.44–1.48	0.96	1.03	0.35–3.00	0.78	1.17	0.40–3.36
≥ 2		0.16	1.63	0.83–3.22	0.62	2.06	0.12–36.00	0.85	0.90	0.29–2.77
None		Referent			Referent			Referent		

*CI* confidence interval, *OR* odds ratio

^a^Education level in ART practitioners was classified into “Below bachelor”, “Master”, and “PhD”. PhD was set as the referent

^b^Occupation status in ART practitioners was classified into “Male-fertility specialist”, “Female-fertility specialist”, “Laboratory technicians” and “Nurse”. Nurse was set as the referent

^c^Professional level in ART practitioners was classified into “Primary”, “Secondary”, “Vice-senior”, and “Senior”. Senior was set as the referent

^d^Marital status in IVF couples was classified into “First married”, and “Remarried”. Remarried was set as the referent

^*^Significant items,* P* < 0.05

### Intracouple agreement and the ability to predict their spouse preferences toward PAR

Whether the attitudes of the surviving spouses could represent the actual willingness of the deceased was explored by comparing the choice predicted by husbands/wives with the actual choices of their wives/husbands. Among the included participants, the prediction accuracies of husbands and wives were 57.87% (125/216, Table 3) and 61.12% (132/216, Table 4), respectively. The interrater agreement analysis showed that unsatisfactory kappa values were found both in the prediction accuracy of husbands (kappa value: 0.338) and wives (kappa value: 0.408). Over half (112/216, 51.85%, Table 5) of wives showed approvals for PAR, while the rate was lower in husbands (39.81%, *X*^2^ = 7.09, *P* = 0.03, Table 5). Intracouple agreement analysis showed that the agreement rate within couples was 65.28% (Table 5). When the participants were asked “whether the attitudes are consistent in important decision-making within couples” (Question 17 in the second questionnaire), 243 of 432 participants (56.25%) agreed that their decisions were always the same, which was slightly lower than the actual prediction rate of males.

**Table 3:** Accuracy of males in predicting their spouse preference for PAR

Female’s attitude	Male’s prediction^a^		
	Destroy	Donate for research	For PAR
Destroy	43 (19.90%)	2 (0.93%)	13 (6.02%)
Donate for research	12 (5.55%)	19 (8.80%)	15 (6.94%)
For PAR	30 (13.89%)	19 (8.80%)	63 (29.17%)

^a^The accuracy rate of males in predicting their spouse preference for PAR was just 57.87% and the kappa index was 0.338

**Table 4:** Accuracy of females in predicting their spouse preference for PAR

Male’s attitude	Female’s prediction^a^		
	Destroy	Donate for research	For PAR
Destroy	50 (23.15%)	8 (3.70%)	22 (10.19%)
Donate for research	17 (7.87%)	26 (12.04%)	7 (3.24%)
For PAR	5 (2.31%)	25 (11.57%)	56 (25.93%)

^a^The accuracy rate of females in predicting their spouse preference for PAR was just 61.12% and the kappa index was 0.408

**Table 5:** Intercouple agreement on actual selections of the posthumous embryos

	Destroy	Donate for research	For PAR
Male’s attitude	80 (37.04%)	50 (23.15%)	86 (39.81%)
Female’s attitude	58 (26.85%)	46 (21.30%)	112 (51.85%)
Intercouple agreement^a^	43 (19.91%)	29 (13.43%)	69 (31.94%)

^a^*X*^2^ = 7.09, *P* = 0.029, The agreement rate between couples was 65.28%

## Discussion

To our knowledge, this is the first study to assess the attitudes toward PAR in the Chinese population. Compared with previous studies [2, 15, 16] from other countries or regions, this study has a larger sample size, and it is the most comprehensive assessment. The results suggest that most people among the included participants approved of PAR in certain situations.

### Changes in the traditional Chinese reproductive viewpoint

Confucianism is the mainstream belief system of modern Chinese society and one of the most crucial spiritual assets in the 5000 years of Chinese culture, which has affected many generations of Chinese people. Following the Confucian influence, an old Chinese saying goes, “Of three forms of unfilial behaviors, the worst is to have no descendants”. Having descendants has been the most critical reproductive viewpoint in China under the influence of Confucianism. Traditionally, Chinese people believe that only a son can pass on the family line. However, the traditional viewpoint has been changed nowadays in China (Fig. 2). This change is partially related to the increase in educational level in China as a result of building the most extensive higher education system globally. The gross enrollment rate for university education has reached 51.6% [17], showing that China has popularized higher education [18]. Participants with higher education showed a more open-mind reproductive viewpoint (Table 2), believing that having children is not obligatory and that boys and girls should be equal in intergenerational transmission.

The “one-child policy”, promulgated by the Chinese government in 1971, may be related to the changing in reproductive viewpoints of Chinese people. In the past 40 years, each family could have only one child. This generation of only children has become the main population in current Chinese society, with a new reproductive viewpoint influenced by the restrictive family planning policy. Some families are are characterized as “double income no kids” (DINKs). It is reported that the proportion of DINK households in the urban areas of Shanghai rose from 4% in 1994 to 12.4% in 2002. Nowadays, 30% of the post-80 s generation agreed with DINK [19]. In conclusion, some traditional Chinese reproductive viewpoints have changed over generations with the initiation of national policy, education extension, lifestyle changes, and economic development.

### The influencing factors of PAR attitudes

Supporting PAR was positively correlated with young participants and married individuals, consistent with the previous research [15]. However, gender was also found to be an factor influencing attitudes toward PAR. Supporting PAR seems easier for females because they are mainly involved in pregnancy, having more and stronger feelings about pregnancy and childbirth. Traditional Confucianism emphasizes the family identity of females as reproductive roles and their responsibility to bear and rear children. In addition, the difference in PAR acceptance between males and females is due to the complexity of PAR after the death of the female partners, as male partners need surrogates or another female partner to carry the pregnancy. Since the lowest education level of ART practitioners was college degrees, which is already in the high education group, there is no significant difference in the education of ART practitioners. Education was no longer the core factor affecting people’s attitudes toward PAR. As for occupation, a significant difference was only reflected in ART practitioners rather than in the general public and IVF patients. A possible reason is that ART practitioners maintain a different degree of contact and emotion with patients or embryos in various aspects. Hence, ART practitioners with different identities have different attitudes toward PAR. Female specialists could understand the fertility needs of patients and the highly valuable through their contact with IVF couples, making them to be more inclined to help patients achieve their reproductive needs. The correlations between income levels and PAR attitudes were inconsistent in different populations. The working relationship or income levels may be affected by IVF treatment. Many IVF patients were forced to work part-time or even quit their jobs due to the treatments, leading to inconsistent results [20, 21]. The manner of conception showed a significant difference only in the general public because of only 27 participants who had conceived via ART were included, accounting for 2.5% of the participants with a pregnancy history. The relatively broad 95% CI value partially reflects the bias in the data. A larger number of participants with a history of ART may have reversed this result. Therefore, the demographic characteristics need to be fully considered before implementing PAR.

### Willingness of the deceased or his/her spouse—which should be first considered?

The PAR approval rates of the three groups were relatively high with the consent of deceased spouse (Fig. 3). Among the included participants, 42.59% of IVF patients approved the use of posthumous embryos for PAR by their spouses without prior explicit consent, which was lower than recent similar study [22]. Peoples who suggested that the willingness of the living spouses should come first believed that the initial purpose of acquiring embryos was to deliver a child, and the spouses had a firm willingness and desire to have children. Therefore, it seemed reasonable to allow spouses to use posthumous embryos for PAR unless there was other evidence indicating that the deceased would have opposed it [23]. However, more than 20% of IVF patients disagreed with using embryos for PAR after death, suggesting that the uncertainty of individuals desiring to have children before death does not represent a willingness to have children after death. In addition, attitudes toward PAR were not always concordant within IVF couples (65.28% consistency rate, Table 5). Therefore, the surviving spouses requiring to initiate PAR may not always represent the desires of the deceased spouse. As a result, in a situation without explicit consent before death, PAR is detrimental to the reproductive autonomy of the deceased. The execution of the reproductive autonomy of the deceased after death [24] is most important. In the absence of consent, PAR should be more implemented more cautiously after weighing the interests and rights of each party [7].

### Rights and healthy growth of offspring

PAR artificially creates single-parent children or even orphans [25]. Undoubtedly, these children grow up with greater mental stress even greater than that of traditional single-parent children. Questions about how PAR affects the posthumous offspring emotionally and psychologically have emerged [26, 27]. A negative effect is that children will feel wronged or stigmatized due to being conceived after one genetic parent died [10]. Another concern is that offspring may consider themselves to be memorial children or replacements for the deceased. Single-parent children miss many classes, have learning disabilities, and suffer attention deficit disorders [28, 29]. In addition, they have worse physical health and material resources than the children of married parents [28, 30, 31]. Furthermore, the extreme situation of the genetic parent’s death highlights the insecurity of the legal rights and welfare of the offspring. Without a stable, warm and supportive parent–child relationship, these children receive less family care, less welfare, and more social pressure. The inheritance rights of posthumous children are debatable, given that the genetic parent may have had no intention for PAR [2]. The inheritance of children born before the parent’s death may be decreased due to the children being born via PAR, raising problems in inheritance rights [32]. However, the offspring of PAR play no role in the decision to initiate their creation. Therefore, they seem to deserve equal benefits that any child is entitled to after the death of a parent [10]. It is critical to have adequate counseling and trauma healing before PAR [33] to ensure responsible reproduction rather than impulsive decisions. Children should not be born as substitutes for others, and their welfare and legal rights after birth should be equal to those of double-parent children.

### Study limitations

Some limitations exist in this study. First, the number of IVF couples, ART practitioners, and participants with below-college education were relatively low. Second, to ensure authenticity and validity and prevent communication and discussion between couples, the survey of IVF couples was only conducted in one reproductive center, resulting in geographical limitations and some bias. Some research bias may be generated through the recruiting method. A national and wider investigation is necessary. Finally, considering the belief of most participants that offspring growth is more important than family inheritances, further studies on the physical and mental health of offspring are expected. More suggestions are needed from psychological and sociological experts to address this issue.

## Conclusions

PAR is a controversial topic involving multiple aspects of psychology, ethics, morality, and law. This study confirmed that the approval rates toward PAR among the three groups of the included participants were generally high. The prediction accuracy and intracouple consistency were moderate. Traditional Chinese attitudes toward reproduction changed essentially. Moreover, the psychological well-being of the offspring should be considered before the implementation of PAR. Appropriate legal policies or specialized guidance in PAR need to be considered and published in China. This study provides some advice and evidence in PAR practice and policy development for medical professionals and policymakers. Due to the very large regional and demographic differences in China, investigations with larger samples of participants are needed.

## Supplementary Information

**Additional file 1.** Instructions for participants.

**Additional file 2.** Questionnaire on public.

**Additional file 3.** Questionnaire on IVF couples.

**Additional file 4.** Questionnaire on ART practitioners.

**Additional file 5: Fig. S1.** Province Distribution of Public Participants.

**Additional file 6: Fig. S2.** Province Distribution of IVF Practitioners.

## Data Availability

Original data can be available from the corresponding author when needed.

## Abbreviations

PAR: Posthumous assisted reproduction
ASRM: The American Society for Reproductive Medicine
ESHRE: The European Society of Human Reproduction and Embryology
ART: Assisted reproductive technology
CSRM: The Chinese Society of Reproductive Medicine
DINKs: Double income no kids
