# Supplementary material for: Attitudes toward posthumous assisted reproduction in China: a multi-dimensional survey
Source: Reprod Health. 2022 May 21;19:122. doi: 10.1186/s12978-022-01423-9 (PMC9124412; doi:10.1186/s12978-022-01423-9)
Supplement: Supplementary file 1 — Additional file 1. Instructions for participants. [file 12978_2022_1423_MOESM1_ESM.docx]

**Instructions for participants**

Dear ladies and gentleman，

We are the Reproductive Research Team of the First Affiliated Hospital of Shantou University Medical College.

In recent years, assisted reproductive technology have become a usual and effective treatment for infertility couples. It makes more and more frozen embryos existed in reproductive centers all over the country. With the prolonged freezing time, the problem that how to deal with the remaining frozen embryos becomes complicated and difficult when a family structure changes (such as one or both accidental death). However, there is no relative legislation and ethical guidelines on posthumous assisted reproduction issues.

Our research team designed this study and desired to measure public, IVF couples and ART staff attitudes toward posthumous reproduction and hope to provide more reasonable recommendations to improve the management of frozen embryos.

This study just collects some attitudes towards some specific situations by anonymous method. We invite you to fill out this questionnaire, which will take you about 3-8 minutes. It will not any burden and have no influence on your treatment. The research does not add any potential risks and injuries to you and your family. The information you filled will just only used for this research. We will keep your personal data confidential. Surely, you can decide whether or not to participate in this questionnaire and quit out of this study at any time.

If you have any questions about this questionnaire or have any suggestions for this study, please do not hesitate to contact us. Thank you for your participation and cooperation.

**Research Contact:** Dr. Jiliang Huang

**Tel：**13790833143

**E-mail：**frank_whuang@126.com

Reproductive Research Team of SUMC
