# Supplementary material for: Attitudes toward posthumous assisted reproduction in China: a multi-dimensional survey
Source: Reprod Health. 2022 May 21;19:122. doi: 10.1186/s12978-022-01423-9 (PMC9124412; doi:10.1186/s12978-022-01423-9)
Supplement: Supplementary file 2 — Additional file 2. Questionnaire on public. [file 12978_2022_1423_MOESM2_ESM.docx]

**Posthumous assisted reproduction questionnaire on public population**

Posthumous assisted reproduction (PAR) refers to the use of gametes or embryos to initiate conception after the death of a genetic parent. Such as the survived wife demand to transfer the frozen embryos after his husband accidental died, or their parents request to take the embryos out for surrogating when both spouses died.

***Part I*** *This section mainly investigates the participants' personal information, and aims to identify the individual influencing factors of the participants' attitudes and decisions (the layered statement is not shown to the participants).*

1. What is your gender?

A. Male B. Female

2. What is your age? ________

3. What is your highest degree?

A. Junior and below B. Senior C. College or Bachelor D. Postgraduate

4. What is your occupation?

A. Unemployed B. Businessman C. office staff D. Professional staff

5. How much is your monthly salary (*yuan*)?

A. ￥3000 or less B. ￥3000-6000 C. 6000-9000 D. 9000 or above

6. Do you have any religious beliefs?

A. Buddhism B. Christianism C. others D. None

7. What is your census register?

A. Rural B. Urban

***Part II*** *This part mainly investigates the participants' marriage and childbearing information, the purpose is to clarify the differences of attitude and decision-making among different reproductive history groups.*

8. What is marital status?

A. Married B. Unmarried

9. How long is your marriage? (*The unmarried skip this question*)

A. 1 year or less B. 1-4 years C. 4-7years D. 7 years or above

10. Have you ever had a history of conception with your spouse? (*The unmarried skip this question*)

A. Yes B. No

11. Have you ever had babies with your spouse? (*if no conception history, skip this question*)

A. one B. two or more C. Never

12. What is the method of conception? (*if no conception history, skip this question*)

A. Natural B. Assisted reproduction

***Part III*** *This section mainly investigates* *the reproductive concepts, the purpose is to understand whether the Chinese traditional concepts changes.*

13. Having a child is an essential thing for a family, do you think so?

A. Yes B. No C. No Opinion

14. Only boys can inherit their family blood, do you think so?

A. Yes B. No C. No Opinion

15. An adopted child can also inherit their family blood, do you think so?

A. Yes B. No C. No Opinion

***Part IV*** *This section mainly investigates the attitude towards PAR issues.*

16. What do you think of the legal characteristics of gametes and embryos?

A. As a potential life B. As one’s property

C. A special substance between life and things D. No Opinion

17. Who do you think has the right to dispose of frozen embryos or gametes in the hospital when one of the couple died accidentally?

A. The hospital B. their spouse C. their parents D. No Opinion

18. Who do you think has the right to dispose of frozen embryos or gametes in the hospital when the couple both died accidentally?

A. The hospital B. their parents C. No Opinion

19. Whether do you think that the surviving spouse requests to use the frozen embryos for posthumous reproduction should be allowed?

A. Yes B. No C. No Opinion

20. Whether do you think that their parents request to use the frozen embryos for posthumous reproduction when the couple both died accidentally should be allowed?

A. Yes B. No C. No Opinion

21. Should the surviving spouse be allowed to continue using frozen embryos for pregnancy in the absence of a written documentation from the deceased?

A. Yes B. No C. No Opinion

22. If PAR is allowed, do you think whether it is necessary to allow adequate time for grieving?

A. Yes B. No C. No Opinion

23. If adequate time is requested for grieving, how long do you think is suitable?

A. 0.5-1 year B. 1-2 years C. more than 2 years D. No Opinion

24. Inheriting their family blood and ensuring offspring healthy grow-up, which do you think is more important?

A. Inheriting family blood B. offspring healthy grow-up C. No Opinion

25. Whether do you think that picking out eggs or sperm after death for posthumous reproduction should be allowed?

A. Yes B. No C. No Opinion
