# Supplementary material for: Attitudes toward posthumous assisted reproduction in China: a multi-dimensional survey
Source: Reprod Health. 2022 May 21;19:122. doi: 10.1186/s12978-022-01423-9 (PMC9124412; doi:10.1186/s12978-022-01423-9)
Supplement: Supplementary file 3 — Additional file 3. Questionnaire on IVF couples. [file 12978_2022_1423_MOESM3_ESM.docx]

**Posthumous assisted reproduction questionnaire on IVF couples**

Posthumous assisted reproduction (PAR) refers to the use of gametes or embryos to initiate conception after the death of a genetic parent. Such as the survived wife demand to transfer the frozen embryos after his husband accidental died, or their parents request to take the embryos out for surrogating when both spouses died.

***Part I*** *This section mainly investigates the participants' personal information, and aims to identify the individual influencing factors of the participants' attitudes and decisions (this layered statement is not shown to the participants)*

1. What is your gender?

A. Male B. Female

2. What is your age? ________

3. What is your highest degree?

A. Junior and below B. Senior C. College or Bachelor D. Postgraduate

4. What is your occupation?

A. Unemployed B. Businessman C. office staff D. Professional staff

5. How much is your monthly salary (*yuan*)?

A. ￥3000 or less B. ￥3000-6000 C. 6000-9000 D. 9000 or above

6. Do you have any religious beliefs?

A. Buddhism B. Christianism C. others D. None

7. What is your census register?

A. Rural B. Urban

***Part II*** *This section mainly investigates* *This part mainly investigates the participants' marriage and childbearing information, the purpose is to clarify the differences of attitude and decision-making among different reproductive history groups.*

8. Are you first married or remarried?

A. First B. remarried

9. How long is your marriage?

A. 1 year or less B. 1-4 years C. 4-7years D. 7 years or above

10. How long has your couple been infertile?

A. 1 year or less B. 1-4 years C. 4-7years D. 7 years or above

11. Have you ever had a conception with your spouse?

A. Yes B. No

12. Have you ever had babies with your spouse?

A. one B. two or more C. Never

13. What is the method of conception? (*if no conception history, skip this question*)

A. Natural B. Assisted reproduction

***Part III*** *This section mainly investigates* *the reproductive concepts, the purpose is to understand whether the Chinese traditional concepts changes.*

14. Having a child is an essential thing for a family, do you think so?

A. Yes B. No C. No Opinion

15. Only boys can inherit their family blood, do you think so?

A. Yes B. No C. No Opinion

16. An adopted child can also inherit their family blood, do you think so?

A. Yes B. No C. No Opinion

***Part IV*** *This section mainly investigates the consistency of attitude between husband and wife in decision-making events and the ability of mutual prediction of decisions made by the spouses.*

17. How about the consistency of attitudes between your couple on important decision-making events?

A. Always B. Sometimes C. Occasionally D. Never

18. The surviving spouse or the died, whose wish do you think is more important?

A. The deceased B. The spouse C. No Opinion

19. In the event of your death, how do you want to deal with the remaining embryos?

A. Destroy B. Donate for scientific research C. The spouse for PAR

20. In the event of your death, how do you think your spouse will to deal with the remaining embryos?

A. Destroy B. Donate for scientific research C. for PAR

***Part V*** *This section mainly investigates the attitude towards PAR issues.*

21. Whether do you think that the surviving spouse requests to use the frozen embryos for posthumous reproduction should be allowed?

A. Yes B. No C. No Opinion

22. Whether do you think that their parents have the right to dispose of the remaining frozen embryos when the couple both died accidentally should be allowed?

A. Yes B. No C. No Opinion

23. Should the surviving spouse be allowed to continue using frozen embryos for pregnancy in the absence of a written documentation from the deceased?

A. Yes B. No C. No Opinion

24. If PAR is allowed, do you think whether it is necessary to allow adequate time for grieving?

A. Yes B. No C. No Opinion

25. If adequate time is requested for grieving, how long do you think is suitable?

A. 0.5-1 year B. 1-2 years C. more than 2 years D. No Opinion

26. Inheriting their family blood or ensuring offspring healthy grow-up, which do you think is more important?

A. Inheriting family blood B. offspring healthy grow-up C. No Opinion

27. Whether do you think that picking out eggs or sperm after death for posthumous reproduction should be allowed?

A. Yes B. No C. No Opinion
