# Supplementary material for: Attitudes toward posthumous assisted reproduction in China: a multi-dimensional survey
Source: Reprod Health. 2022 May 21;19:122. doi: 10.1186/s12978-022-01423-9 (PMC9124412; doi:10.1186/s12978-022-01423-9)
Supplement: Supplementary file 4 — Additional file 4. Questionnaire on ART practitioners. [file 12978_2022_1423_MOESM4_ESM.docx]

**Posthumous assisted reproduction questionnaire on ART practitioners**

Posthumous assisted reproduction (PAR) refers to the use of gametes or embryos to initiate conception after the death of a genetic parent. Such as the survived wife demand to transfer the frozen embryos after his husband accidental died, or their parents request to take the embryos out for surrogating when both spouses died.

***Part I*** *This section mainly investigates the participants' personal information, and aims to identify the individual influencing factors of the participants' attitudes and decisions (this layered statement is not shown to the participants)*

1. What is your occupation?

A. Andrologist B. Gynaecologist C. Nurse D. IVF-lab staff

2. What is your age? ________

3. What is your highest degree?

A. College B. Bachelor C. Master D. PhD

4. What is your professional tittle?

A. Junior B. Senior C. associate professor D. professor

5. which city are you in? ________

***Part II*** *This section mainly investigates* *This part mainly investigates the participants' marriage and childbearing information, the purpose is to clarify the differences of attitude and decision-making among different reproductive history groups.*

6. What is marital status?

A. Married B. Unmarried

7. Have you ever had a history of conception with your spouse? (*The unmarried skip this question*)

A. Yes B. No

8. Have you ever had babies with your spouse? (*The unmarried skip this question*)

A. one B. two or more C. Never

9. What is the method of conception? (*if no conception history, skip this question*)

A. Natural B. Assisted reproduction

***Part III*** *This section mainly investigates the attitude towards PAR issues.*

10. What do you think of the legal characteristics of gametes and embryos?

A. As a potential life B. As one’s property

C. A special substance between life and things D. No Opinion

11. Who do you think has the right to dispose of frozen embryos or gametes in the hospital when one of the couple died accidentally?

A. The hospital B. their spouse C. their parents D. No Opinion

12. Who do you think has the right to dispose of frozen embryos or gametes in the hospital when the couple both died accidentally?

A. The hospital B. their parents C. No Opinion

13. Should the surviving spouse be allowed to continue using frozen embryos for pregnancy in the absence of a written documentation from the deceased?

A. Yes B. No C. No Opinion

14. Should the surviving spouse be allowed to continue using frozen embryos for pregnancy with a written documentation from the deceased?

A. Yes B. No C. No Opinion

15. Is it fair of being forced to destroy the frozen embryo when one of the couple died accidentally?

A. Yes B. No C. No Opinion

16. whether is it necessary to allow PAR in China?

A. Yes B. No C. No Opinion

17. Inheriting their family bloodline and offspring healthy grow-up, which do you think is more important?

A. Family bloodline B. offspring healthy grow-up C. No Opinion

18. do you think whether it is necessary to allow adequate time for grieving?

A. Yes B. No C. No Opinion

19. If adequate time is requested for grieving, how long do you think is suitable?

A. 0.5-1 year B. 1-2 years C. more than 2 years D. No Opinion
