# Supplementary figures and images for: Attitudes toward posthumous assisted reproduction in China: a multi-dimensional survey
Source: Reprod Health. 2022 May 21;19:122. doi: 10.1186/s12978-022-01423-9 (PMC9124412; doi:10.1186/s12978-022-01423-9)

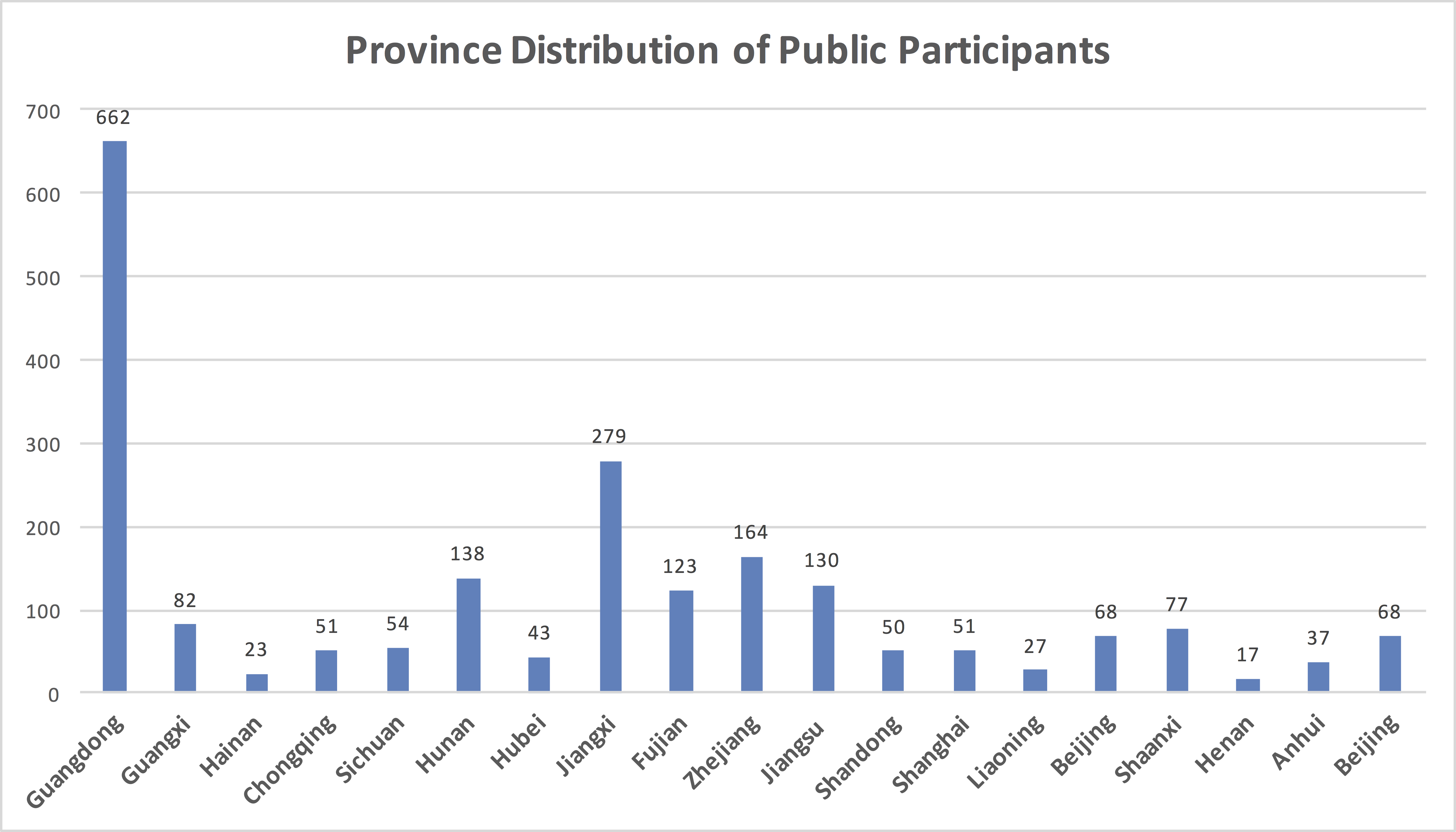

Supplement: Supplementary file 5 — Additional file 5: Fig. S1. Province Distribution of Public Participants. [file 12978_2022_1423_MOESM5_ESM.tiff]

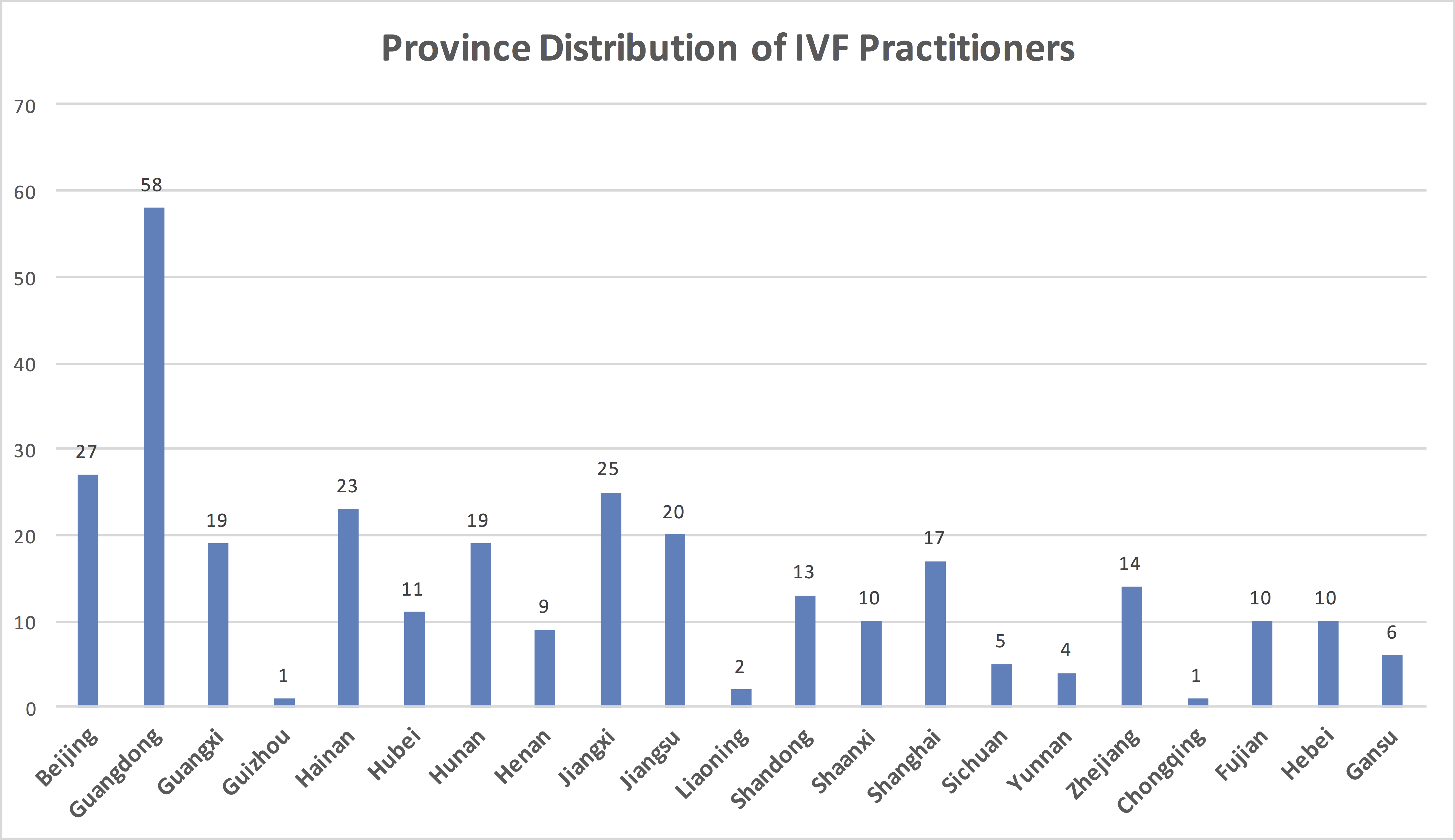

Supplement: Supplementary file 6 — Additional file 6: Fig. S2. Province Distribution of IVF Practitioners. [file 12978_2022_1423_MOESM6_ESM.tiff]
